# Laminin Receptor in Shrimp Is a Cellular Attachment Receptor for White Spot Syndrome Virus

**DOI:** 10.1371/journal.pone.0156375

**Published:** 2016-06-03

**Authors:** Wang-Jing Liu, Yi-Chieh Li, Guang-Hsiung Kou, Chu-Fang Lo

**Affiliations:** 1 Department of Earth and Life Science, College of Science, University of Taipei, Taipei, Taiwan; 2 Department of Life Science, College of Life Science, National Taiwan University, Taipei, Taiwan; 3 Institute of Bioinformatics and Biosignal Transduction, College of Bioscience and Biotechnology, National Cheng Kung University, Tainan, Taiwan; Uppsala University, SWEDEN

## Abstract

White spot syndrome virus (WSSV, genus *Whispovirus*, family *Nimaviridae*) is causing huge economic losses in global shrimp farming, but there is no effective control. Shrimp cell laminin receptor (Lamr) may have a role in WSSV infection. The objective was to characterize interactions between *Penaeus monodon* Lamr (PmLamr) and WSSV structural proteins. In this study, PmLamr interacted with nine WSSV structural proteins (based on yeast two-hybrid screening), of which one (VP31) was characterized. Protein pull-down assay confirmed the interaction between PmLamr and VP31; the latter was an envelope protein exposed outside the WSSV virion (based on membrane topology assays). Furthermore, similar to mammalian Lamr, there were two major protein bands in shrimp cells. Cellular localization assay demonstrated VP31 co-localized with PmLamr on transfected cells. Enzyme-link immunosorbent assay (ELISA) and competitive ELISA demonstrated binding of VP31 on PmLamr was dose-dependent; however, addition of WSSV virion competed for binding affinity. Furthermore, based on an *in vivo* neutralization assay, both VP31 and PmLamr delayed mortality in shrimp challenged with WSSV. We concluded Lamr was an important receptor for WSSV infection and the viral envelope protein VP31 may have a role in host cell recognition and binding. These data contributed to elucidating pathogenesis of WSSV infection and may help in controlling this disease.

## Introduction

White spot syndrome virus (WSSV; family *Nimaviridae*, genus *Whispovirus* [1]) is a double-stranded DNA virus, which contains a large genome (~307 kbp). This virus affects most cultured shrimp with cumulative morality approaching 100% within 3 to 7 d after disease onset [2–5]. WSSV has an extensive host range (> 93 species of arthropods are known hosts or carriers [5]) and based on wide tissue tropism, the cellular receptor for WSSV is predicted to be conserved and ubiquitous [6]. Various proteins have been suspected to mediate WSSV infection, including *Penaeus monodon* Rab7 (PmRab7) [7], *P*. *monodon* chitin-binding protein (PmCBP) [8], beta-integrin [6], F1 ATP synthase beta subunit [9], or glucose transporter 1 [10, 11].

Laminin receptor (Lamr), which is a cell surface receptor, is notable because it mediates high-affinity interactions between laminin and the cell. Lamr has a predicted molecular mass of 32 kDa. However, when in SDS-polyacrylamide gels it is found to have an apparent electrophoretic mobility of ~37 kDa, and further processed into a 67-kDa protein [12]. Based on its molecular weight and function, Lamr has been designated 37/67-kDa laminin receptor (37LR, 67LR, LAMR1), 32 kDa laminin binding protein (LBP), 32 kDa laminin binding protein precursor (LBP-32, 37LRP), p40 and ribosomal protein SA (RPSA) [13]. Furthermore, Lamr has been also recognized as a multifunctional protein involved in not just cell adhesion, but also a wide range of biological processes, such as cell development, mobility and differentiation [14]. Moreover, it has also been reported that Lamr acts as a receptor for several exogenous agents, including prion proteins, viruses and bacteria [13]. In shrimp, Lamr was first identified as a receptor protein for Taura syndrome virus (TSV) [15]. It was subsequently reported to act as a binding protein for two additional shrimp RNA viruses (infectious myonecrosis virus [IMNV] and yellow head virus [YHV]) [16] and was also implicated in hemocyte homeostasis for white shrimp, *Litopenaeus vannamei* [17]. In the present research, the potential role of shrimp Lamr in WSSV infection was investigated. Results indicated that PmLamr may act as a host cellular receptor which bound to the WSSV envelope protein VP31 and mediated WSSV infection.

## Materials and Methods

### PmLamr cloning and expression in yeast

Protein-protein interaction assays were performed by using the Matchmaker Gold yeast two-hybrid system (Clontech). These assays were carried out in order to identify candidates of WSSV structural proteins that could interact with *P*. *monodon* Lamr (PmLamr). By cloning the PCR-amplified cDNA fragment encoding PmLamr (Genbank accession number DT044263) into the pGBKT7 vector (Clontech) in frame with the GAL4 DNA binding domain (DBD), the bait plasmid pGBKT7-PmLamr was produced. The resulting bait plasmid was then transformed into yeast (*Saccharomyces cerevisiae*) strain Y2HGold. The expression of GAL4 DBD-PmLamr fusion bait protein was confirmed by Western blotting with an anti-c-Myc antibody (Abcam). The autoactivation and toxicity of the bait protein was tested before two-hybrid screening. Sequences of primers used for pGBKT7-PmLamr construction are listed (Table 1).

**Table 1:** Primers used for generating plasmids used in this study.

Plasmid	Strand	Sequences (5’-3’)
pGBKT7-PmLamr	Forward	CCGGAATTCATGTCGGGAGGATTAAGTGTT ^a^
	Reverse	TGCACTGCAGTTACCAGTTGGATCCATCAGC
pET28b/PmLamr-His	Forward	CCGGAATTCGATGTCGGGAGGATTAAGTGTTATG
	Reverse	CCCAAGCTTCCAGTTGGATCCATCAGCTGCGCC
pMAL/VP31	Forward	CCGGAATTCTATGAATGAGAGCTTACAGCTG
	Reverse	TCCCCGCGGCGCACGGGAAGTAACGCT
pDHsp/PmLamr-V5-His	Forward	CCGGAATTCTATGTCGGGAGGATTAAGTGTT
	Reverse	TCCCCGCGGCCAGTTGGATCCATCAGCTGC

^a^Restriction enzyme cutting sites are underlined.

### Yeast two-hybrid assay

By cloning the 49 WSSV structural protein and three *P*. *monodon* cellular protein genes, respectively, into the vector pGADT7 (Clontech) and then transforming the resulting plasmids into the *S*. *cerevisiae* host strain Y187, a prey library was produced [18]. In order to identify PmLamr interaction candidate proteins, the prey library clones were mated with the bait (i.e. the pGBKT7-PmLamr-transformed Y2HGold). Both positive and negative controls were made, and this was done by mating pGADT7-T-transformed Y187 prey with pGBKT7-53- or pGBKT7-Lam-transformed bait, respectively (the manufacturer provided the corresponding plasmids). A minimal synthetically defined (SD) double-dropout (DDO; SD medium lacking Leu and Trp [SD/-Leu/-Trp]) medium supplemented with 5-bromo-4-chloro-3-indolyl-α-D-galactopyranoside (X-α-Gal) and Aureobasidin A (DDO/X/A) was used to select positive clones which expressed prey proteins that interacted with PmLamr (bait). Blue colonies that were found to be growing on a DDO/X/A medium were subsequently patched onto higher stringency quadruple-dropout (QDO; SD medium without Ade, His, Leu, and Trp [SD/-Ade/-His/-Leu/-Trp]) plates containing X-α-Gal and Aureobasidin A (QDO/X/A).

### Expression and purification of native PmLamr-His and antibody production

The cDNA fragment encoding PmLamr was cloned into the pET-28b(+) vector (Novagen), resulting in plasmid pET28b/PmLamr-His. Sequences of primers used for pET28b/PmLamr-His construction are listed (Table 1). To express a His-tagged-PmLamr (PmLamr-His) fusion protein, pET28b/PmLamr-His was transformed into *Escherichia coli* strain BL21-CodonPlus (DE3)-RIL (Stratagene), and protein expression induced with 0.1 mM isopropylthiogalactoside (IPTG) overnight at 16°C. Following centrifugation (6,000 ×g for 10 min), *E*. *coli* cell pellets were re-suspended in lysis buffer (50 mM Tris-HCl [pH 7.4], 300 mM NaCl, 20 mM imidazole, 1 mM PMSF, 0.25 mg/ml lysozyme) and sonicated on ice. Cellular debris was removed by centrifugation (10,000 ×g for 30 min). Ninitriloacetic acid (Ni-NTA) beads (Qiagen) were added to the supernatant and rotating mixing at 4°C for 2 h, then washed with washing buffer (50 mM Tris-HCl [pH 7.4], 300 mM NaCl, and 100 mM imidazole). Thereafter, PmLamr-His was eluted with elution buffer (50 mM Tris-HCl [pH 7.4], 300 mM NaCl and 250 mM immidazole). Fractions of PmLamr-His were collected, pooled and dialyzed against 1× PBS and used in antibody preparation, pull-down assays, enzyme-link immunosorbent assays (ELISA) and *in vivo* neutralization assays. For polyclonal antibody production, the fusion protein–PmLamr-His was subjected to SDS-PAGE; protein bands containing fusion proteins were sliced from the gel, minced, mixed with Freund’s complete adjuvant, and inoculated into rabbits.

### Expression and purification of native MBP or MBP-VP31

An MBP tagged VP31 (WSSV T-1, GenBank accession number AF440570) expression construct–pMAL/VP31 was formed by digesting the amplified VP31 gene fragment using EcoRI and SacII and ligating it into the corresponding restriction enzyme cutting sites of pMAL-c5X (NEB). Sequences of primers used for pMAL/VP31 construction are listed (Table 1). An excess of recombinant MBP or recombinant MBP-VP31 was produced in *E*. *coli* strain BL21-CodonPlus (DE3)-RIL with 0.1 mM IPTG incubated overnight at 16°C. Following centrifugation (6,000 ×g for 10 min), *E*. *coli* cell pellets were re-suspended in lysis buffer (20 mM Tris-HCl [pH 7.4], 200 mM NaCl, 1 mM EDTA, 1 mM DTT, 1 mM PMSF, 0.25 mg/ml lysozyme, and 0.1% Triton X-100), kept on ice and sonicated. Cellular debris was removed by centrifugation (10,000 ×g for 30 min). Then the supernatant was added to amylose resins (NEB) and rotating mixing at 4°C for 2 h and washed with washing buffer (20 mM Tris-HCl [pH 7.4], 200 mM NaCl, 1 mM EDTA, 1 mM DTT). Finally, MBP and MBP-VP31 were eluted with elution buffer (washing buffer with 10 mM maltose). Fractions were collected, pooled and dialyzed against 1× PBS. These proteins were used for the following pull-down, ELISA, and *in vivo* neutralization assays.

### Interaction of MBP-VP31 with PmLamr-His (protein pull-down assay)

Approximately 500 ng of PmLamr-His was incubated with 1 μg of either MBP or MBP-VP31 bound to amylose resins in 150 μl TBST buffer (10 mM Tris-HCl [pH 7.2], 150 mM NaCl, 1% Triton X-100, 10 mM EDTA, and a protease inhibitor cocktail tablet [Roche]) at 4°C for 3 h. After seven 10-min washes with TBST buffer, proteins that bound to the beads were resolved by 12% SDS-PAGE followed by Western blot analysis. The presence of PmLamr-His was detected using anti-PmLamr polyclonal antibody and goat anti-rabbit IgG-horseradish peroxidase (HRP) conjugate secondary antibody (Sigma). Protein band signals were visualized using a chemiluminescence reagent ECL (Perkin-Elmer).

### Membrane topology of VP31

The membrane topology of VP31 predictions were predicted with TMHMM (http://www.cbs.dtu.dk/services/TMHMM-2.0/; [19]) and HMMTOP (http://www.enzim.hu/hmmtop/) [20, 21] based on the deduced aa sequence of VP31. Both methods were used in single-sequence mode, with all user-adjustable parameters set at default values. To further investigate the membrane topology of VP31 in the WSSV virion, experiments were done as described [22]. For a duration of 2 h at 37°C, aliquots (5 μg of total protein) of purified virions were treated with 5 μg/ml trypsin (Promega) in 100 μl of buffer (50 mM Tris-HCl [pH 7.5], 1 mM CaCl_2_, 100 mM NaCl). Triton X-100 (final concentration, 1%) was added in some samples prior to trypsin digestion, and this was done in order to dissolve the viral envelope and expose internal structures to trypsin. For the purpose of analyzing samples, Western blotting using antibodies against VP31 [23] was implemented, and with the aim of facilitating comparisons it was also implemented against tegument protein VP26 and the envelope protein VP28. The present study involves a WSSV virion; healthy crayfish (*Procambarus clarkii)* were challenged with WSSV-T1 strain and virus purified for the purpose of preparing the WSSV virion. Please see the description in Xie and Yang [24].

### Protein lysates preparation and Western blot analysis

Shrimp stomach tissues were lysed in 0.33× PBS with protein inhibitor cocktail, centrifuged at 12,000 ×g for 5 min, and supernatant retained for Western blot analysis. To prepare PmLamr recombinant proteins from *Drosophila* S2 cells, the full-length PmLamr coding region was inserted into a heat-inducible *Drosophila* heat shock protein 70 promoter (pDHsp/V5-His [25]) by PCR cloning (using primers listed in Table 1 to generate pDHsp/PmLamr-V5-His). For DNA transfection, *Drosophila* S2 cells were seeded onto 24-well plates (8 × 10^5^ cells/well) and grown in Schneider’s medium (Invitrogen) supplemented with 10% fetal calf serum at 28°C. After 1 h, the pDHsp/PmLamr-V5-His plasmid (200 ng of plasmid DNA per well) was transfected into the S2 cells with Effectene reagent (Qiagen) for 16 to 18 h, followed by heat shock (37°C in a water bath for 30 min). At 72 h after transfection, S2 cells were lysed in 25 μl NP-40 lysis buffer (50 mM Tris-HCl [pH 8.0], 150 mM NaCl and 1% NP-40). Cell lysates were separated by 12% SDS-PAGE, followed by Western blotting. PmLamr proteins were detected using an anti-PmLamr polyclonal antibody.

### Confocal microscopy and co-localization

*Drosophila* S2 cells were seeded onto glass coverslips placed in the wells of a 24-well plate and transfected with pDHsp/PmLamr-V5-His plasmid, as described above. After 48 h, S2 cells were incubated with 1 μg of either MBP or MBP-VP31 for 2 h at 28°C. Attached cells were washed twice with PBS, fixed with freshly prepared 4% formaldehyde in PBS for 10 min and then washed with PBS two times. After blocking with PBS containing 5% bovine serum albumin and 2% normal goat serum for a duration of 16 h at 4°C, the cells were then incubated for a total of 3 h at room temperature (RT) with either the 1:500 PBS-diluted rabbit anti-PmLamr antibody, the 1:500 PBS-diluted mouse anti-MBP antibody (NEB), or with both antibodies. Next, the cells were washed a total of three times with PBST (PBS containing 0.2% Tween-20), and then reacted for 2 h at RT with either 1:1000 PBS-diluted carboxymethylindocyanine (Cy3) dye-conjugated goat anti-rabbit IgG antibody (Sigma), 1:1000 PBS-diluted Alexa Fluor^®^ 488 dye-conjugated goat anti-mouse IgG antibody (Invitrogen), or with both. A confocal microscope (Leica TCS SP5) was used to detect fluorescence signals.

### Binding specificity of PmLamr-His to MBP-VP31 (ELISA and competitive ELISA)

For ELISA, flat-bottomed 96-well ELISA plates (Corning Costar) were coated with 2 μg PmLamr-His (in 0.1 M bicarbonate/carbonate buffer [pH 9.6]; 100 μl per well) and incubated overnight at 4°C. Plates were then washed three times with washing buffer (1× PBS containing 0.05% Tween-20), and blocking buffer (washing buffer with additional 1% BSA) was added, followed by incubation for 2 h at RT. After washing with washing buffer, various dilutions of MBP or MBP-VP31 (0 to 400 ng) in blocking buffer were added to plates, followed by incubation for 2 h at RT. Plates were washed and a blocking buffer diluted detection antibody (mouse anti-MBP antibody) was added (100 μl per well), followed by incubation for 1 h at RT. After washing, HRP-labeled goat anti-mouse antibody (Santa Cruz) was added to plates, and reaction mixtures incubated for an additional 1 h at RT. The reaction was visualized using the HRP substrate TMB (3,3’,5,5’-tetramethylbenzidine; eBioscience), stopped by adding 100 μl 1 N H_2_SO_4_. An ELISA reader (biochrom) was used to read the absorbance immediately at 450 nm. For competitive ELISA, 96-well ELISA plates were first coated with 2 μg PmLamr-His, followed by washing and blocking. Thereafter, 200 ng of MBP-VP31 mixed with various concentrations (0 to 1200 ng) of WSSV viral proteins or BSA were added to plates, and bound MBP-VP31 detected with anti-MBP antibody (as described above). Each experiment was done twice (three wells each time).

### *In vivo* neutralization of WSSV with the MBP-VP31 and PmLamr-His

Batches of *L*. *vannamei* (~3 g of body weight) were used for *in vivo* neutralization assays. The *L*. *vannamei* were bought from National Taiwan Ocean University’s Aquatic Animal Center, and then before experiments were started for at least 3 to 5 d the shrimp were acclimatized in water tanks with a salinity of 33 ± 1 ppt at 25 ± 1°C. The inoculum used was prepared from WSSV-T1 strain, as described [26]. For *in vivo* neutralization, shrimp were allocated into five groups (20 shrimp in each group). First, a constant volume (50 μl) of 10^−7^ dilution of original WSSV inoculum was mixed with 4 μg of MBP-VP31, PmLamr-His, or MBP, for 1 h on ice. These mixtures were used to challenge *L*. *vannamei* (intramuscular injections). Groups of shrimp injected with WSSV (10^−7^ viral diluent) mixed with MBP or 1× PBS buffer served as positive and negative controls, respectively. Shrimp were examined twice daily for 11 d and mortalities recorded. The Kaplan-Meier plot (log-rank test) was used to compare mortality.

## Results

### PmLamr interacted with nine WSSV structural proteins

**Fig 1:**
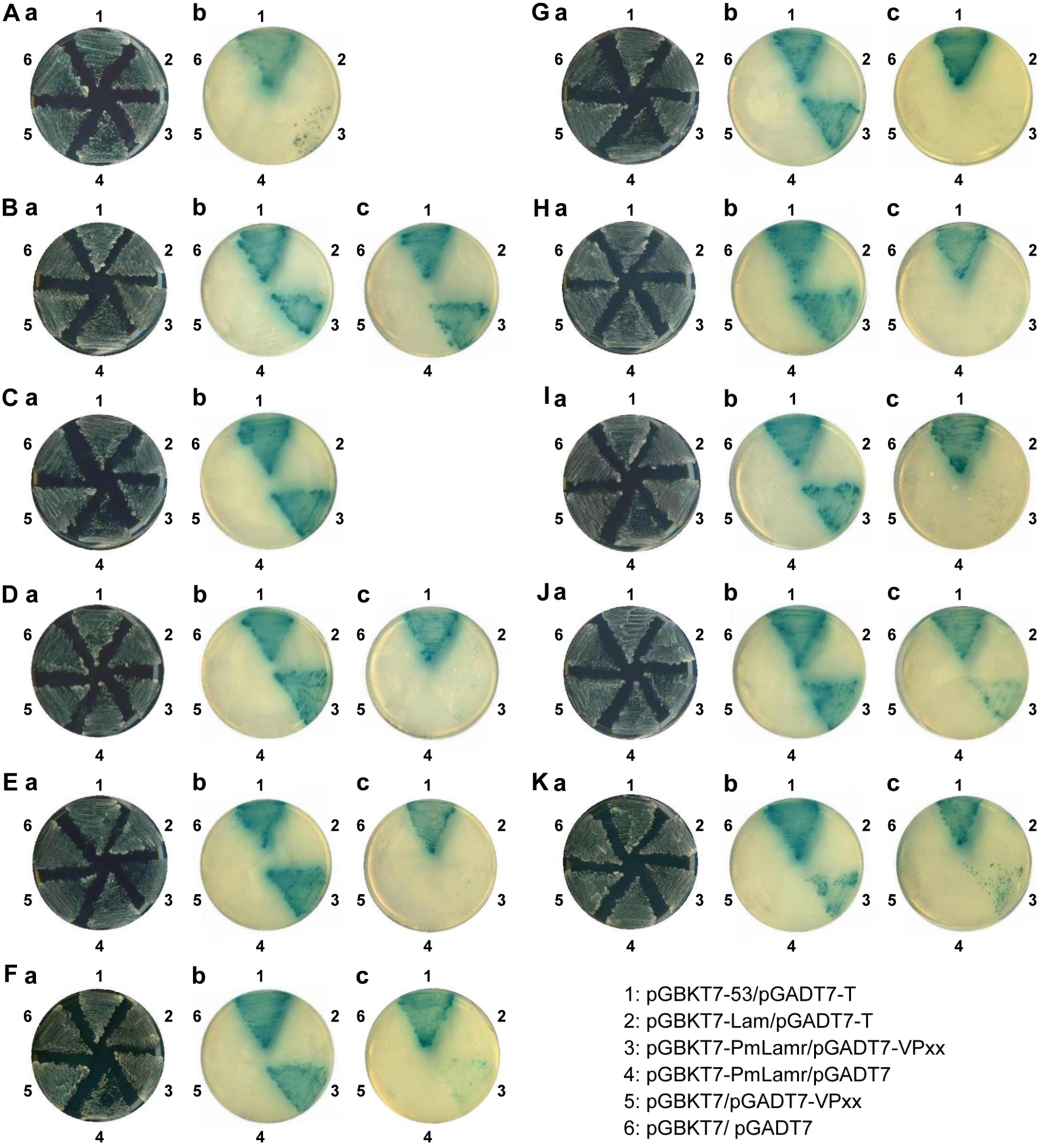
Yeast two-hybrid screening results for interactions between PmLamr and nine WSSV structural proteins. (A) to (K) PmLamr’s interactions with VP16, VP19, VP31, VP38B, VP41A, VP41B, VP52B, VP150-N, VP150-C, VP187-N, and VP187-C, respectively. (a) Yeast grows on types of medium that lack both Leu and Trp (DDO), and this reveals the presence of each respective pair of plasmids. (b) and (c) indicate that yeast grows on low stringency (DDO/X/A) as well as high stringency (QDO/X/A) medium. Blue signals are seen in both (b) and (c) because of the presence of X-α-Gal. Protein-protein interactions are represented by yeast growth, whereas numbers around plates indicate the transformed yeast’s bait and prey plasmids: 1, pGBKT7-53/pGADT7-T; 2, pGBKT7-Lam/pGADT7-T; 3, pGBKT7-PmLamr/pGADT7-VPxx; 4, pGBKT7-PmLamr/pGADT7; 5, pGBKT7/pGADT7-VPxx; 6, pGBKT7/pGADT7, with VPxx respectively, representing each of the WSSV structural proteins listed above.

An auto-activation test for GAL4 DBD fused PmLamr found that in the absence of prey protein, reporter genes in Y2HGold did not autonomously activate. GAL4 DBD fused PmLamr was also not found to be toxic to yeast cells (data not shown). Therefore, it was used as bait to screen the prey library. Yeast two-hybrid mating detected nine WSSV structural proteins (Fig 1) that interacted with PmLamr, including VP16, VP19, VP31, VP38B, VP41A, VP41B, VP52B, VP150-N (N-terminal fragment [aa 1–651] of VP150), VP150-C (C-terminal fragment [aa 641–1302] of VP150), VP187-N (N-terminal fragment [aa 1–804] of VP187), and VP187-C (C-terminal fragment [aa 810–1607] of VP187).

### PmLamr interacted with VP31 in a protein pull-down assay

**Fig 2:**
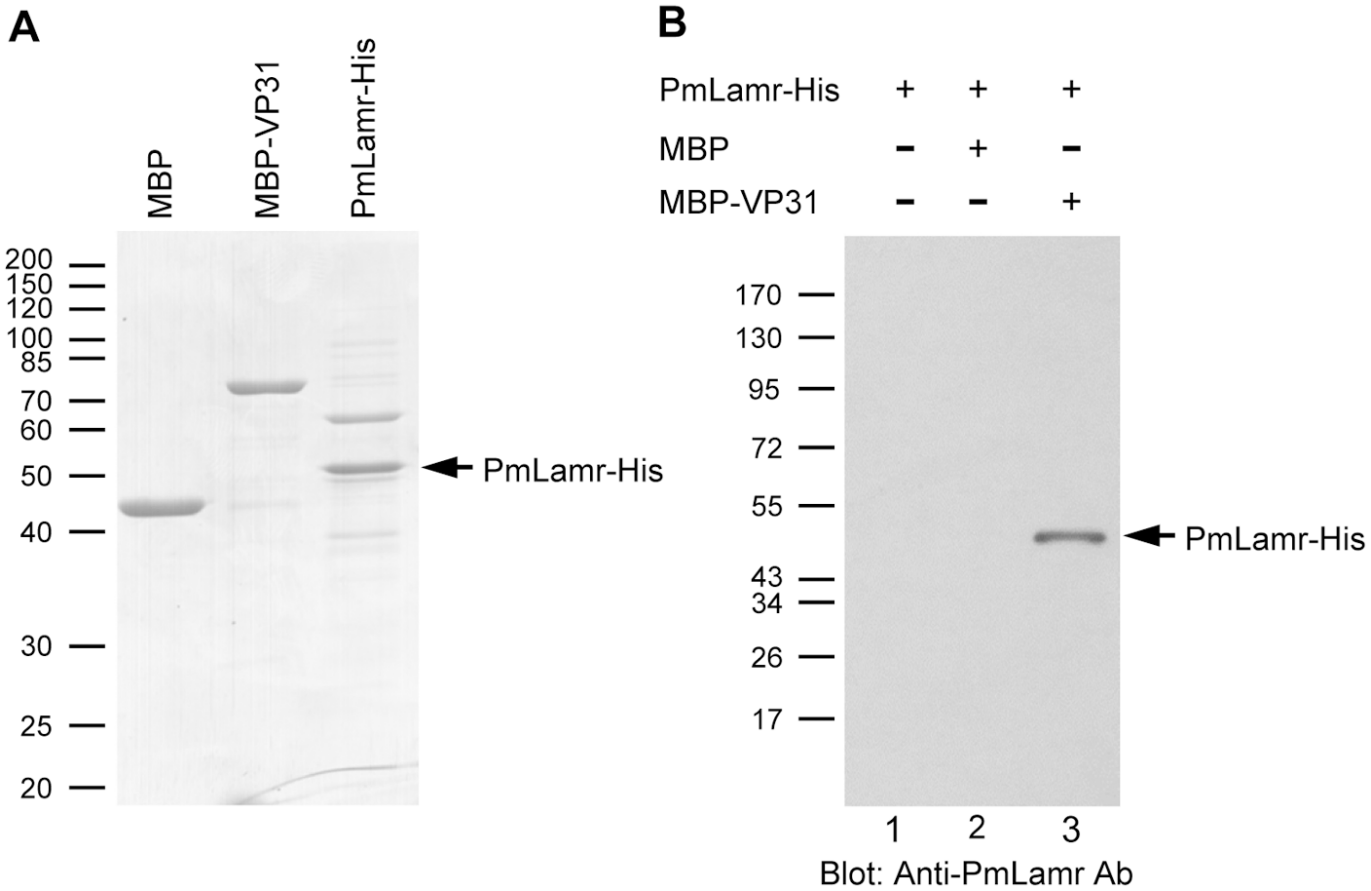
PmLamr interacted with WSSV VP31. Protein pull-down assays of PmLamr-His with MBP-VP31. (A) SDS-PAGE of purified MBP, MBP-VP31 and PmLamr-His proteins. (B) PmLamr-His was incubated with amylose resins (Lane 1), MBP- (Lane 2), or MBP-VP31- (Lane 3) conjugated to amylose resins, pelleted, washed, and detected by immunoblotting (anti-PmLamr antibody).

One of the PmLamr interaction proteins identified by yeast two-hybrid screening was VP31, reported as having an important role in interactions with host cells and contributing to viral pathogenesis [27–29]. Furthermore, it was also involved in recognition of integrin [30] and chitin-binding protein [31]. To further confirm interactions between PmLamr and VP31, a pull-down assay followed by Western blotting analysis using anti-PmLamr antibody was performed. All recombinant proteins for pull-down assays were successfully expressed (Fig 2A). The PmLamr-His was pulled down by MBP-VP31, but not by amylose resins nor MBP (Fig 2B; compare Lane 3 with Lanes 1 and 2), confirming that VP31 bound to shrimp receptor Lamr.

### Membrane topology of VP31

**Fig 3:**
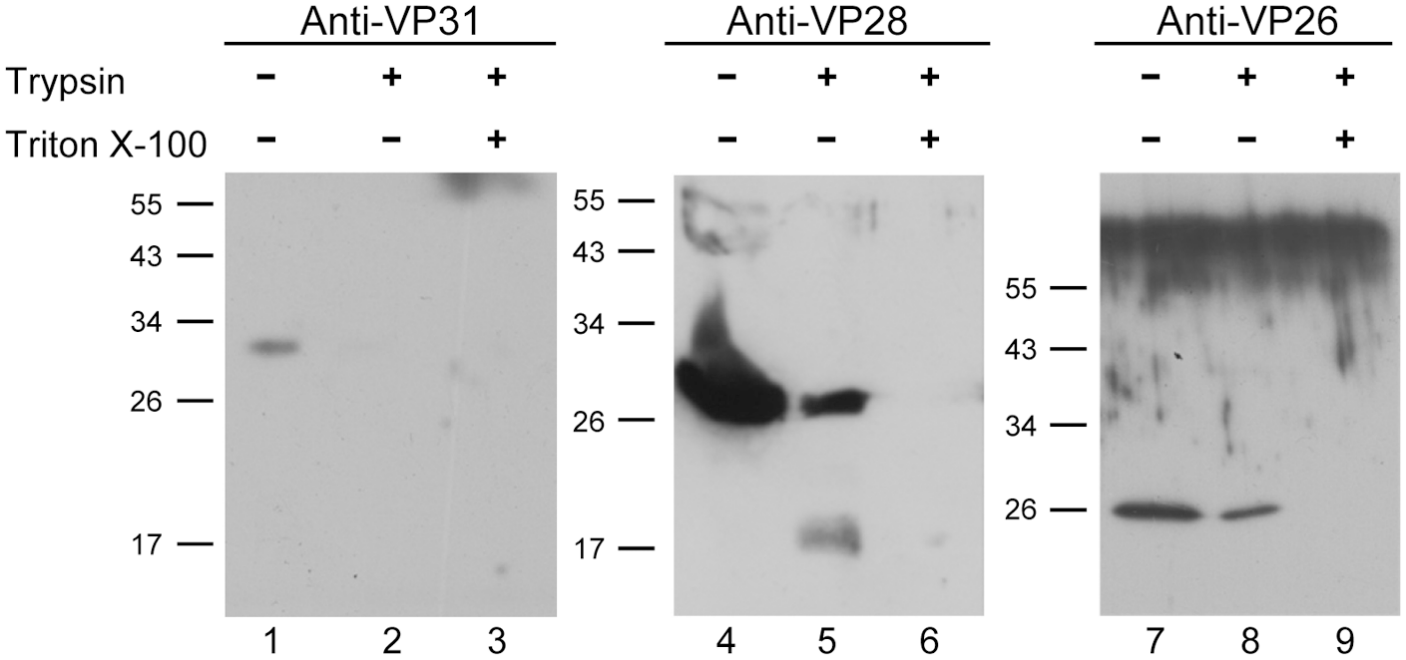
Membrane topology of WSSV VP31. Western blot analysis using corresponding antibodies showing trypsin digestion of VP31 (Lanes 1–3), and two other proteins are compared: VP28 (an envelope protein where most of its C-terminal is located on the envelope’s exterior; Lanes 4–6) and VP26 (a tegument protein; Lanes 7–9) in both intact (Lanes 2, 5 and 8) and detergent-treated virions (Lanes 3, 6 and 9). Lanes 1, 4 and 7: intact virion with neither trypsin nor Triton-X100 treatment.

Two approaches (TMHMM and HMMTOP) to predict VP31 membrane topology yielded very similar results: VP31 was predicted to lack transmembrane helices and to be located outside the envelope. To confirm the predictions concerning the topology of VP31, the WSSV virions were subjected to trypsin digestion in order to distinguish proteins that were accessible to proteolysis and those that were protected from digestion because of the lipid bilayer. The WSSV virions were either left untreated or treated with trypsin in the presence or absence of the detergent Triton X-100. Then, the digested products were analyzed with Western blotting, which used antibodies against VP31, VP26, or VP28. As anticipated, in untreated virions, anti-VP31 antibody recognized the VP31 proteins (Fig 3, Lane 1). However, the 31 kDa protein was no longer detected after trypsin digestion, regardless of whether it was in the presence or absence of Triton X-100 (Fig 3, Lanes 2 and 3). For comparison purposes, in order to detect the VP26 (a tegument protein, which is protected from trypsin digestion by the envelope) and the VP28 (an envelope protein with a portion of its C-terminal exposed outside the virion), the treated virions were also subjected to Western blotting. In the absence of Triton X-100, VP28 was digested into two bands, and in the presence of Triton X-100 it was completely digested. The small band was the transmembrane region of VP28 that was protected by envelope from trypsin digestion (Fig 3, Lanes 4–6). Furthermore, VP26 was digested only in the presence of Triton X-100, as it was not digested in the absence of Triton X-100 (Fig 3, Lanes 7–9).

### Molecular mass of PmLamr proteins

**Fig 4:**
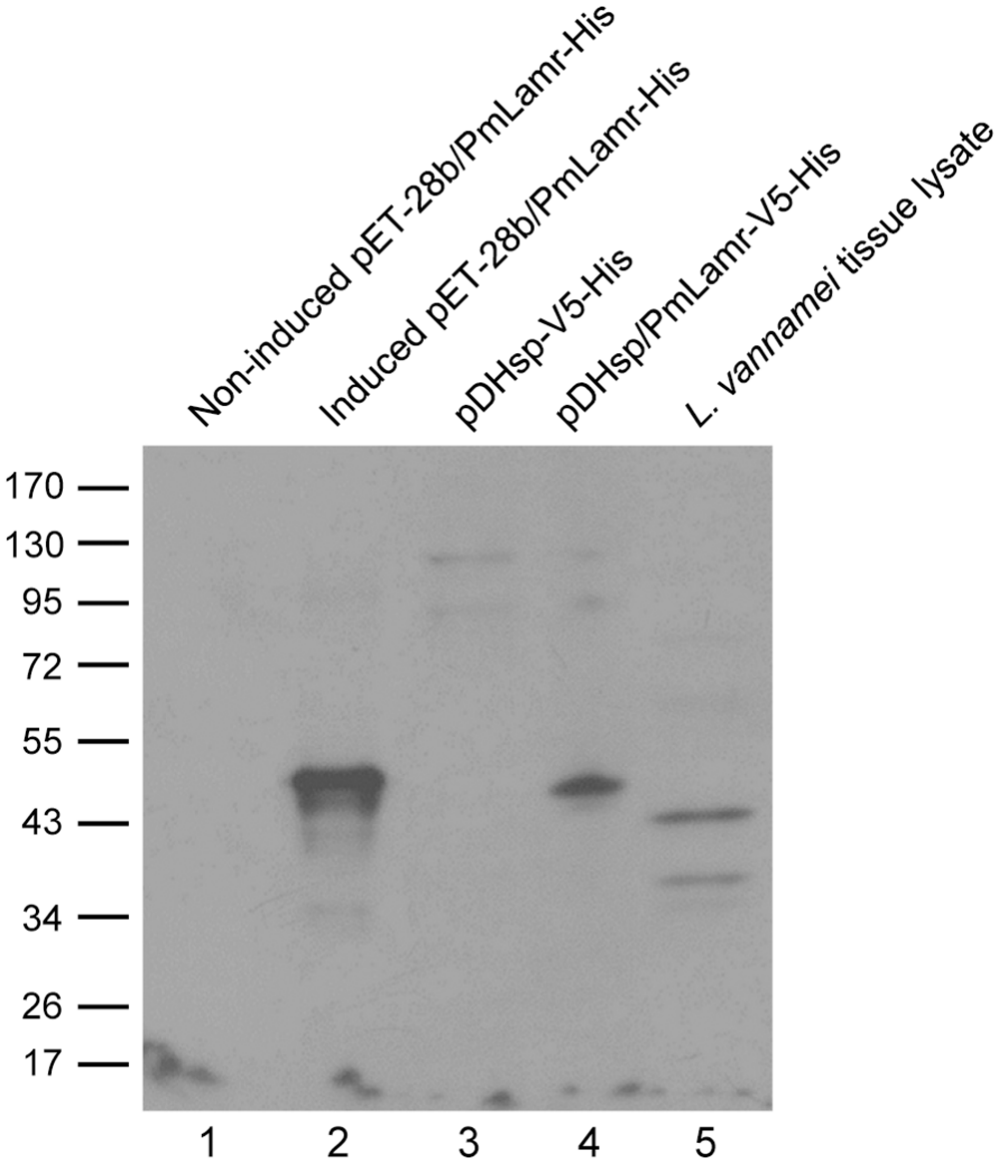
Molecular mass of shrimp Lamr and recombinant PmLamrs expressed in various expression systems. Western blot analysis of lysates of pET-28b/PmLamr-His transformed *E*. *coli* cells, without or with induction of protein expression (Lanes 1 and 2, respectively), pDHsp-V5-His (empty vector) or pDHsp/PmLamr-V5-His transfected *Drosophila* S2 cells (Lanes 3 and 4, respectively), and *L*. *vannamei* stomach tissue (Lane 5), using anti-PmLamr antibody.

Using an antibody derived from the full-length PmLamr coding region, Western blot analysis was performed on the shrimp tissue proteins and recombinant proteins of *E*. *coli* and insect cells. The molecular mass of PmLamr overexpressed in the *E*. *coli* and in *Drosophila* S2 cells was ~51 kDa (Fig 4, Lanes 2 and 4). The Lamr between *P*. *monodon* and *L*. *vannamei* shared 97% identity in amino acid sequences [32]. Western blot analysis also successfully detected two major bands (37 and 44 kDa, respectively) in *L*. *vannamei* stomach tissue lysates, using antibody derived from recombinant PmLamr (Fig 4, Lane 5).

### Co-localization of PmLamr with VP31 on the surface of *Drosophila* S2 cells

**Fig 5:**
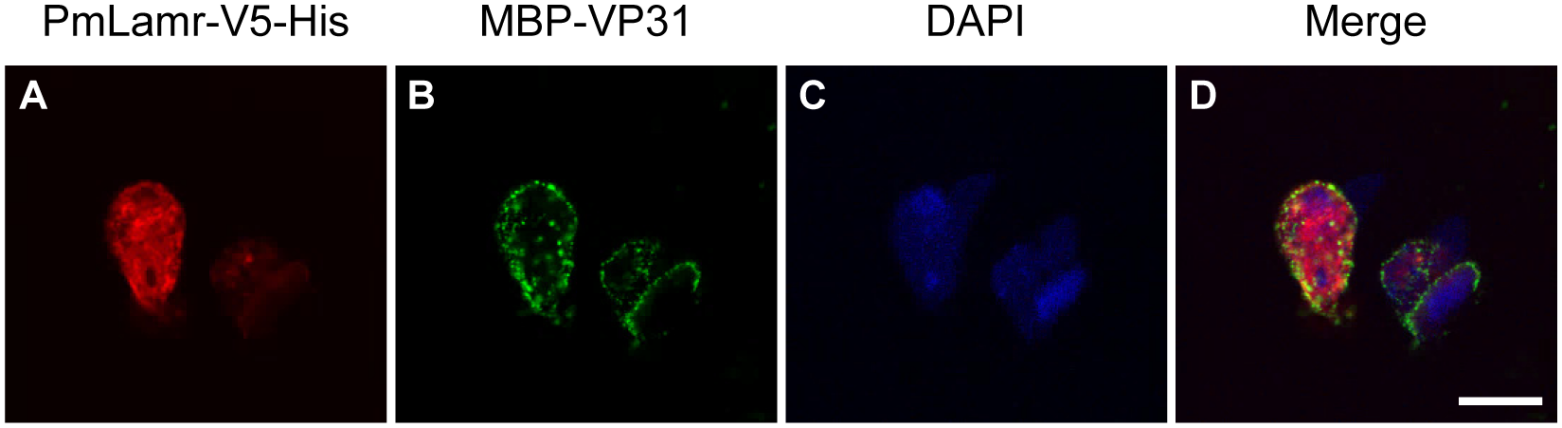
Immunofluorescence images; note colocalization of PmLamr-V5-His with MBP-VP31 on cells. *Drosophila* S2 cells were transfected with pDHsp/PmLamr-V5-His. At 48 h after transfection, 1 μg of MBP-VP31 was added. The presence of (A) PmLamr-V5-His (red) is visualized by rabbit anti-PmLamr antibody and Cy3 dye-conjugated goat anti-rabbit IgG antibody. As for (B) MBP-VP31 (green), mouse anti-MBP antibody and Alexa Fluor^®^ 488 dye-conjugated goat anti-mouse IgG antibody were used. (C) The nuclear DNA was counterstained by DAPI. (D) Merged Cy3, Alexa Fluor^®^ 488, and DAPI signals. Scale bar = 10 μm.

Immunofluorescence analysis of non-permeabilized *Drosophila* S2 cells employing PmLamr- (Fig 5A) and MBP-specific (for detection of MBP-VP31; Fig 5B) antibodies demonstrated that PmLamr and VP31 co-localized on the surface of these cells (Fig 5D). When recombinant protein MBP was added to PmLamr-transfected *Drosophila* S2 cells, no Alexa Fluor^®^ 488 dye signal was produced (control; data not shown).

### VP31 and PmLamr binding specificity determined by ELISA and competitive ELISA

**Fig 6:**
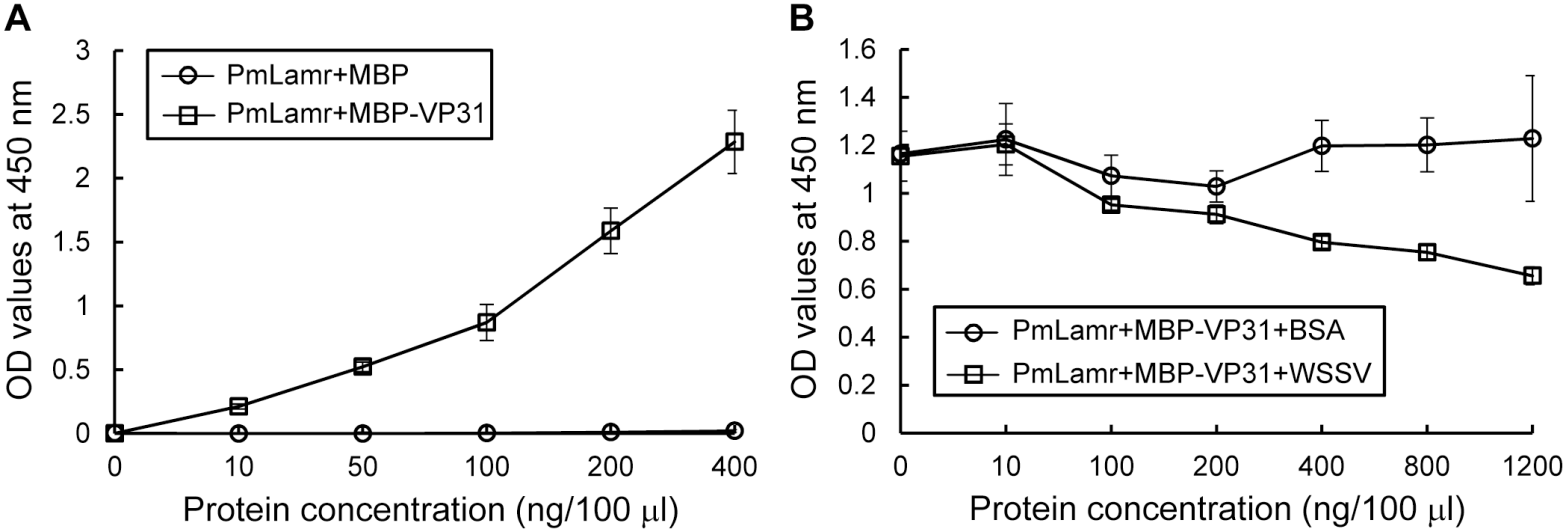
ELISA and competitive ELISA assays demonstrated WSSV VP31 binding to the PmLamr was specific. (A) ELISA analysis of MBP-VP31 binding to the PmLamr-His in a dose-dependent manner. PmLamr-His (2 μg/ well) was coated to a 96-well plate and incubated with various amounts of purified MBP-VP31. MBP was added as negative control. (B) In competitive ELISA analysis, binding of MBP-VP31 and PmLamr-His were competed by WSSV virion proteins. PmLamr-His (2 μg/well) was coated to a 96-well plate. MBP-VP31 (200 ng) with various amounts of purified WSSV virion proteins (10–1200 ng) were added to wells and incubated. Addition of BSA (10–1200 ng) to wells served as a negative control. Mean (± SD) from three replicates.

Increasing MBP-VP31 protein concentration in ELISAs enhanced ability to bind to PmLamr-His (Fig 6A), although there was no binding between MBP and PmLamr-His (control; Fig 6A). Therefore, MBP-VP31 binding to the PmLamr-His was specific and dose-dependent. In order to further clarify the relationship between VP31 and WSSV virion binding to PmLamr, a competitive ELISA assay was performed with MBP-VP31 and purified WSSV. When MBP-VP31 was kept constant (200 ng/100 μl), the OD value decreased as concentrations of WSSV viral proteins increased (from 10 to 1200 ng), whereas for control protein (BSA), OD value was approximately constant when BSA concentrations increased (Fig 6B). Therefore, the WSSV virion specifically competed with VP31 to bind PmLamr.

### *In vivo* neutralization

**Fig 7:**
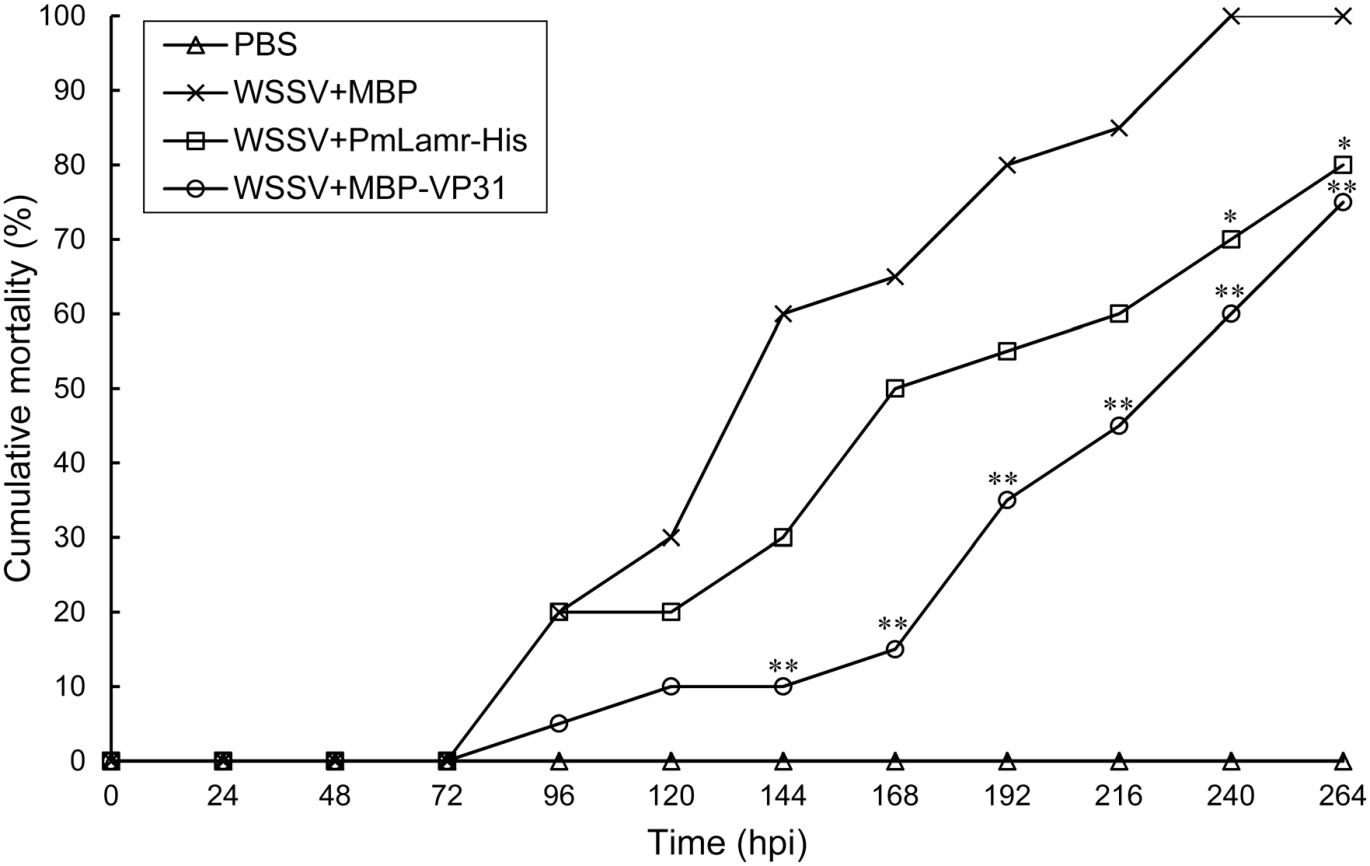
Cumulative post-challenge shrimp mortality. Shrimp were challenged with inoculum containing WSSV mixed with PmLamr-His (WSSV+PmLamr-His), or inoculum containing WSSV mixed with MBP-VP31 (WSSV+MBP-VP31). Asterisks indicated differences between positive and experimental groups (**P<0.01, *P<0.05). Shrimp injected with inoculum containing WSSV mixed with MBP (WSSV+MBP), or injected with PBS only, were positive and negative controls, respectively.

For *in vivo* neutralization experiments using shrimp, with the exception of the PBS vehicle injection group, mortalities in the other three groups of shrimp increased steadily after 72 h post injection (hpi). Furthermore, 100% mortality occurred within 240 hpi in a positive control group (shrimp challenged with inoculum containing WSSV mixed with MBP [WSSV+MBP]). Notwithstanding, cumulative motilities were lower (* P<0.05) than in the positive control group at 240 and 264 hpi for shrimp injected with inoculum containing WSSV mixed with PmLamr-His (WSSV+ PmLamr-His), and at each time point from 144 to 264 hpi (** P<0.01) of shrimp injected with inoculum containing WSSV mixed with MBP-VP31 (WSSV+MBP-VP31; Fig 7). No shrimp mortality occurred in the PBS vehicle injected group, whereas all dead shrimp were WSSV-positive on PCR. Therefore, recombinant WSSV VP31 and PmLamr proteins delayed WSSV infection in shrimp *in vivo*.

## Discussion

Based on outcomes of the present study, we concluded that PmLamr was a receptor for VP31. In that regard, Lamr is a multifunctional protein that acts as a receptor for viruses that cause infection in addition to being involved in a wide range of other biological processes [33]. Furthermore, Lamr of *P*. *monodon* (PmLamr) has been designated as a receptor for three viruses, namely TSV, IMNV and YHV [15, 16]. However, relationships between shrimp Lamr and WSSV, had not been adequately characterized. In the present study, PmLamr interacted with WSSV and mediated infection. In addition, the receptor’s interactions with viral compartments were also dissected. It was noteworthy that PmLamr interacted (yeast two-hybrid screening) with at least nine WSSV structural proteins. Nearly all these interactions were confirmed by co-immunoprecipitation (data not shown), although VP31 was not confirmed, as it was not expressed in insect cells. Regardless, the interaction between VP31 and PmLamr was also confirmed (pull-down assay). Based on neutralization experiments, VP31 was suggested as an important molecule in WSSV infection [27]. Consequently, VP31 was studied in detail.

It was reported that WSSV VP31 protein was an envelope protein (based on virion protein fractionation and immunoelectron microscopy; [22, 23, 26]. The present study confirmed VP31 is an envelope protein. After comparing both information and results concerning the membrane topology of VP31, we inferred that VP31 was an envelope protein, consistent with predictions of TMHMM and HMMTOP software. In the absence of typical transmembrane domains (predicted configurations and the trypsin digestion assay), VP31 may associate with other viral structural protein(s) by forming a complex to anchor on the envelope. Furthermore, interactions with WSSV envelope proteins VP24, VP32, and VP53 may cause VP31 to locate on the virion [31].

The corresponding gene of PmLamr encoded a protein of ~34.1 kDa. In the present study, there were two protein bands (recognized as PmLamr) in shrimp tissue which migrated on SDS-PAGE with a molecular weight of 37 and 44 kDa, respectively. Although the 37 kDa PmLamr band was not yet been reported in shrimp, the 44 kDa band was similar that detected in P. monodon and L vannamei muscle lysates [32]. A similar molecular mass discrepancy was reported in 37LRP/67LR, which was attributed to post-translational covalent modifications or formation of very tightly associated homo- or heterodimers [12, 34], although this is not well characterized.

In addition to yeast two-hybrid and pull-down assays, interactions between VP31 and PmLamr were also identified in cells. For example, in cells that overexpressed PmLamr-V5-His and treated with MBP-VP31, the latter co-localized with PmLamr-V5-His. *In vitro* binding assays further confirmed specificity of interactions between VP31 and PmLamr, whereas recombinant PmLamr and VP31 delayed or neutralized WSSV’s activity, thereby reducing mortality (*in vivo* neutralization assays). Collectively, based on these outcomes, we inferred that Lamr and VP31 had important roles in WSSV infection, namely that PmLamr was a cellular receptor for WSSV attachment and VP31 was involved in recognition of this receptor during virus infection.

It has been reported that Lamr most likely is unable to act as a singular receptor for infection. For example, adeno-associated virus serotype 2 (AAV2) appeared to rely on HSPG for cell attachment. As for efficient internalization and transduction, AAV2 requires Lamr interaction. The idea that there is a synergistic function of the two molecules for AAV2 transduction is further substantiated by evidence that shows Lamr and HSPG can form a complex on the cell surface [35]. In addition, WSSV VP31 was previously suggested as an important molecule involved in WSSV infection [27]. VP31 contained an integrin recognition RGD (conserved Arg-Gly-Asp sequence) motif; this motif was reported to be involved in host cell adhesion and might be associated with beta-integrin [29, 30]. Integrin is another type receptor of laminin. It has been suggested that the large protein 67LR facilitates interactions between laminin and integrins [36]. Furthermore, VP31 can also bind with Lamr and integrin. Binding of VP31 both with shrimp Lamr and integrin suggested that PmLamr may also serve as a co-receptor in shrimp cells to promote interactions between VP31 and integrin, and have a role in pathogenesis of WSSV infection.

Many believe that interactions between cellular receptors and viral attachment proteins are important in determining virus tissue tropism and host range. The WSSV is a virus with wide host range and often causes systemic infection in hosts. Homologues to Lamr were detected in all taxonomic domains of life [13]. In addition, Lamr was widely distributed in shrimp tissues [32]. Therefore, Lamr thus may contribute to the virus’ wide host range and tissue tropism. Elucidating relationships between WSSV components and Lamr should provide further insights regarding pathogenesis of WSSV infections and contribute to development of antiviral strategies for WSSV.
